# The long reach of childhood income inequality: a multinational twin study of gene–environment interplay on adult depressive symptoms

**DOI:** 10.1017/S0033291726104620

**Published:** 2026-06-08

**Authors:** Andrew J. Petkus, Chandra A. Reynolds, Brian K. Finch, Kyla Thomas, Christopher R. Beam, Vibeke S. Catts, Malin Ericsson, Deborah G. Finkel, Carol E. Franz, William S. Kremen, Lisbeth Aagaard Larsen, Nicholas G. Martin, Matt McGue, Miriam A. Mosing, Jenae M. Neiderhiser, Marianne Nygaard, Nancy L. Pedersen, Anbupalam Thalamuthu, Keith E. Whitfield, Margaret Gatz, the IGEMS Consortium

**Affiliations:** 1 Department of Neurology, University of Southern California Keck School of Medicine, Los Angeles, USA; 2 Institute for Behavioral Genetics and Department of Psychology and Neuroscience, University of Colorado Boulder, Boulder, USA; 3 Center for Economic and Social Research and Department of Sociology and Spatial Sciences, University of Southern California, Los Angeles, USA; 4 Center for Economic and Social Research, University of Southern California, Los Angeles, USA; 5 Department of Psychology and Leonard Davis School of Gerontology, University of Southern California, Los Angeles, USA; 6 Centre for Healthy Brain Ageing (CHeBA), Discipline of Psychiatry and Mental Health, School of Clinical Medicine, University of New South Wales, Sydney, Australia; 7 Department of Medical Epidemiology and Biostatistics, Karolinska Institutet, Stockholm, Sweden; 8 Institute for Gerontology, Jönköping University, Jönköping, Sweden; 9 Department of Psychiatry, University of California San Diego, La Jolla, USA; 10 The Danish Twin Registry, Department of Public Health, University of Southern Denmark: Syddansk Universitet, Odense, Denmark; 11 Mental Health and Neuroscience, Queensland Institute of Medical Research – QIMR: QIMR Berghofer Medical Research, Brisbane, Australia; 12 Department of Psychology, University of Minnesota Twin Cities, Minneapolis, USA; 13 Max Planck Institute for Empirical Aesthetics, Frankfurt, Germany; 14 Department of Psychology, The Pennsylvania State University, University Park, USA; 15 Program for Research on Men’s Health, Hopkins Center for Health Disparities Research, Johns Hopkins University Bloomberg School of Public Health, Baltimore, USA

**Keywords:** aged, depressive symptoms, economic inequalities, twin study, diathesis-stress, polygenic index

## Abstract

**Background:**

Living in a country with a large gap between high and low earners has been linked to poor health, including depression. Less studied is gene-by-environment interplay with income inequality as the environmental exposure. Here, we examine the association between childhood exposure to inequality and individual differences in adult depressive symptoms, testing for moderation of genetic influences by inequality using polygenic indices for major depressive disorder, as well as twin models.

**Methods:**

The research participants were 69,924 members of twin studies from four developed countries, born between 1893 and 1979, aged 22–103 years at depressive symptom assessment. Genotyping was available for 6,256 participants. Income inequality was operationalized as share of income accruing to the top 1% for each country when the participants were between age 5 and 15 years.

**Results:**

Childhood income inequality was associated with depressive symptom scores in adulthood, adjusting for covariates. Each 1% rise in inequality was associated with 0.295 higher depressive symptoms (scaled on T-score units). In genetic analyses, interaction effects showed that men who faced more inequality as children and had higher genetic risk for depression reported modestly higher depressive symptoms compared to other men. For women, both genetic risk and inequality mattered, with each independently associated with depressive symptoms. Twin models showed that inequality moderated genetic variance underlying depressive symptoms; heritability of depressive symptoms was higher where exposure to income inequality was higher.

**Conclusions:**

Findings illustrate the long reach of childhood exposure to income inequality and suggest that advantaged environments may help protect against the effects of deleterious genes.

## Introduction

High income inequality has demonstrable individual and societal effects on population health, including mortality, low birthweight, brain structure and function, other morbidities, and depression (Lynch et al., 1998; Messias, Eaton, & Grooms, 2011; Pickett & Wilkinson, 2015; Rakesh et al., 2025a; 2025b). Income inequality is a structural feature of a society and can have negative effects on both the affluent and the poor (Layte et al., 2019; Rakesh et al., 2025a; Subramanian & Kawachi, 2006). Some work takes into account that a society’s overall wealth may shape the resources available to buffer against inequality (Rakesh et al., 2025a), although the nature of this relationship is not well established. One research team, using state-level income inequality data combined with individual-level Behavioral Risk Factor Surveillance System (BRFSS) data, found a significant effect of income inequality (Gini coefficient) on depression that persisted after adjustment for income and social capital (Messias et al., 2011). A subsequent meta-analysis of the association of inequality and elevated depressive symptoms reported a pooled risk ratio of 1.19 (95% CI: 1.07, 1.31) (Patel et al., 2018).

In considering the effects of income inequality on population health, it is increasingly clear that environmental exposures during key developmental periods in childhood may be most relevant for future health (Duncan & Murnane, 2011). A US study, using Panel Study of Income Dynamics (PSID) data, found that stronger food safety nets during adolescence have significant health effects that manifest decades after initial exposure (Hoynes, Schanzenbach, & Almond, 2016). Across different countries, adverse childhood experiences and associated social determinants of health have been studied in relation to adult depression (Daníelsdóttir et al., 2024; Kirkbride et al., 2024). Building on this work, we argue that a country’s income inequality in childhood can serve as an exogenous indicator of early life exposure to adverse social conditions.

Work on inequality, however, rarely accounts for genetic influences on health outcomes and how inequality can moderate the effects of genetic propensity. One exception is a review of the interplay of inequality and heritability for educational outcomes in a population (Selita & Kovas, 2019). As the authors show, environmental disparities may significantly affect the relationship of genetic propensities to individual-level outcomes. Further, Finch et al. (2026) discovered that the protective effect of educational attainment for dementia, and to a lesser extent the genetic predisposition for education, is strongest in contexts where educational inequality is low.

Gene–environment interaction (GxE) occurs when the strength of genetic influence is moderated by environmental circumstance (Boardman, Daw, & Freese, 2013; Reiss, Leve, & Neiderhiser, 2013). The diathesis-stress model predicts that high-risk environments will have greater detrimental impact on individuals who are at elevated genetic risk. Caspi et al. (2003) were one of the first to describe such an interaction: individuals with a particular genetic polymorphism showed greater depressive symptoms when exposed to stressful life events compared with individuals who did not have that polymorphism. While questions have been raised about the replicability of this finding for a candidate gene, there are strong reasons to believe that GxE phenomena are more common when considering polygenic influences (Duncan & Keller, 2011). Studies of GxE typically do not feature exogenous indicators of the environmental factor but often rely on questionnaire-based measures of stressful life events, adverse childhood experiences, and socioeconomic status (e.g. Bleys, Luyten, Soenens, & Claes, 2018; Illius, Eder, Vogel, & Alexander, 2026). Relevant here is that self- or family-reported measures of the environmental exposure may introduce genetic confounding; a threat mitigated by an exogenous indicator of environment.

A further test of whether the strength of genetic influence is moderated by environmental circumstance directly examines moderation of heritability in twin samples. The diathesis-stress model predicts that the importance of genetic factors for disease is maximized in adverse environments and minimized in favorable ones. For example, genetic influences on obesity are greater in adverse than in beneficial environments (Johnson et al., 2011). In turn, heritability would be reduced in advantaged environments where negative genetic propensities are offset by greater resources or more equally distributed resources. It has further been noted that interactions between heritability and various socioeconomic status indicators are observed more often in the United States than in more egalitarian countries (Colodro-Conde et al., 2015; Selita & Kovas, 2019).

Twin studies have shown that depressive symptoms are modestly heritable (Kendler, Gatz, Gardner, & Pedersen, 2006; McGue & Christensen, 2003). Whether these heritability estimates are moderated by environmental factors is not well explored. If genetic expression of depressive symptoms is promoted by unfavorable environments, such a result might contribute to explaining how income inequality is associated with depression.

## Research hypotheses

Our central focus is gene-by-environment interplay in associations between depressive symptoms in mid- to older adulthood and country-level income inequality in childhood.

Our first research question is whether the degree of country-level income inequality to which an individual was exposed in childhood is related to the level of depressive symptoms in adulthood, and whether sex or the country’s overall wealth at that time moderates observed associations. While the research literature suggests that we should find an association between inequality and depressive symptoms, it is necessary to test this question in our data before proceeding to GxE hypotheses.

Our second research question is whether exposure to country-level income inequality in childhood more greatly exacerbates adult depressive symptoms among individuals who are at higher genetic risk for depression based on their polygenic risk scores.

Our third research question asks whether exposure to country-level income inequality in childhood moderates the overall heritability of depressive symptoms in adults by applying twin models to estimate heritability.

## Methods

### Overview of design

To examine Question 1, we employed a large sample of middle-aged and older adult twins, including both men and women, from a multinational consortium. A subset of participants had genotyping information for calculating a polygenic index (PGI), indicating genetic risk for major depressive disorder, allowing us to address Question 2. Inclusion of monozygotic (MZ), dizygotic (DZ), and opposite-sex (OS) twin pairs allowed application of twin models to estimate heritability, enabling us to address Question 3.

We use Top 1%, that is, the share of the country’s income accounted for by the highest 1% earners of its population (representing the gap between high and low earners), as our measure of income inequality due to the availability historically of this measure for the countries included in the analyses (Hasell & Arriagada, 2023). Gini coefficient data are not available for this complete time span. Change in the Top 1% income share is moderately negatively correlated with life expectancy (Kenworthy, 2016), supporting its validity as a measure for studies of health. Because a country’s wealth may moderate the effects of high inequality, we added Gross Domestic Product (GDP; Hasell & Arriagada) as a key moderating variable.

Levels of income inequality vary across countries and over time. Our use of twin samples from different birth cohorts and multiple countries allows us to explore a range of differences in exposure to inequality. If only one country is included, year, income inequality, and GDP are completely confounded. By including multiple countries, different participants may have experienced different levels of exposure to inequality, even in the same year.

## Participants and studies

All samples included in the analyses are part of the Interplay of Genes and Environment in Multiple Studies (IGEMS) consortium (Finkel et al., 2025a, Pedersen et al., 2013; Pedersen et al., 2019). We included 15 studies from four countries (Denmark, Sweden, Australia, and the United States). Each sample was required to have data on depressive symptoms, educational attainment, and income inequality. The combined analytic sample in the main analysis was 69,924 individuals. The subset with polygenic index data included 6,256 individuals.

Detailed descriptions of each participating study are included in the Supplementary Text.

## Measures

### Depressive symptoms

The depressive symptom measure is a harmonized score developed using item response methods from a separate crosswalk sample who responded to the different measures of depressive symptoms from the different participating IGEMS studies (Gatz et al., 2015, and Supplementary Text). For ease of analysis, the harmonized depressive symptom scores were standardized as T-scores.

### Country-level indicators: Top 1%, GDP

Top 1% is the share of the national income accounted for by the highest 1% of a country’s population, indicating inequality in the distribution of income (Hasell & Arriagada, 2023). Gross Domestic Product (GDP) is a widely used standard measure of the value of the production of goods and services in a country during a given year, thus indicating a country’s standard of living at that time. Top 1% and GDP were downloaded from Our World in Data (Hasell & Arriagada, 2023) for each IGEMS country from 1903 through 1989. For analysis, we calculated the mean Top 1% score and GDP by averaging across the years corresponding to the participant’s ages 5–15. This age range serves as a window around age 10, which we designated as childhood with respect to childhood exposure.

### Other covariates

Educational attainment was harmonized across all IGEMS samples based on the International Standard Classification of Education (ISCED) that uses nine categories ranging from less than primary education through graduate degree (UNESCO Institute for Statistics, 2012). ISCED level 3 approximates high school education. Date of birth and sex were obtained from civil registries or by self-report when the twin registries were compiled.

### Polygenic indices (PGIs) for depression

PGIs were computed using the summary statistics from a GWAS of major depressive disorder (MDD; Howard et al., 2019) and applying the SBayesR method with robust option (GCTB 2.03 beta2; Lloyd-Jones et al., 2019) based on the protocol from the IGEMS consortium (IGEMS Consortium, 2022). We retained HapMap3 SNPs (International HapMap 3 Consortium, 2010) that were available across all IGEMS samples, excluding rare (MAF < 1%) or poorly imputed (INFO < 0.8) variants. After obtaining SBayesR weights, all IGEMS studies applied PLINK under one of two versions with perfectly corresponding results (v1.90, Purcell et al., 2007; v2.00a3, Chang et al., 2015). Untyped MZ twins were assigned PGI scores based on their genotyped cotwin, using available zygosity information. Before analysis, PGI scores were adjusted for genetic ancestry using the first 10 principal components. PGI scores were standardized (*M* = 0.0, SD = 1.0) within each IGEMS study sample.

### Statistical analyses

Participant descriptive statistics were generated, followed by the examination of the distribution of all variables of interest, and bivariate correlations (Supplementary Figure S1 presents a bivariate correlation matrix and distributions of childhood inequality, depressive symptoms, and all covariates of interest). Mixed-effects linear regressions were used to examine the phenotypic associations between income inequality and depressive symptoms. To account for twin pair dependency, individuals were clustered within a twin pair. Potential confounders included chronological age in years, sex, and education. Previous IGEMS studies have shown a nonlinear effect of age on depressive symptoms. Therefore, we included a piecewise-linear effect of age in the model centered at age 70 to adjust for the effect of chronological age on depressive symptoms.

Next, we examined whether the effects of income inequality on depressive symptoms were modified by sex, childhood GDP, and attained education. We examined the continuous income inequality by effect modifier interaction term, which was added to the multivariable linear regression for each effect modifier separately. Using multigroup structural equation modeling (SEM), we then estimated the adverse effect of childhood inequality on depressive symptoms stratified by the effect modifier. For sex, we estimated the effect of childhood inequality on depressive symptoms within men and women separately. Childhood GDP was divided into quartiles. Attained education was divided into the categories, less than high school, high school, and more than high school.

Finally, we ran biometric twin models. Twin studies enable the decomposition of variance in an outcome into additive genetic, shared environmental, and nonshared environmental components. MZ twin pairs share 100%, and DZ twins, on average, share 50% of their segregating genes. Therefore, the additive genetic variance is correlated at 1.0 for MZ twin pairs and .50 for DZ twin pairs. We first fit a univariate twin model to estimate the additive genetic, shared environmental, and nonshared environmental contributions to depressive symptoms. We then fit a model in which income inequality was allowed to moderate the paths on the A, C, and E variances. Supplementary Figure S2 provides a depiction of the fitted model. Income inequality and possible confounders, including age and sex, were allowed to moderate the mean. Equal means and variances were assumed within pairs and across zygosity.

A sensitivity analysis was conducted to test the robustness of our findings. Although the income inequality and GDP variables accounted for the country of origin, there may be further country-level confounding factors that were not accounted for. Therefore, we conducted a sensitivity analysis where we examined the main effect of childhood inequality on depressive symptoms while adjusting for country of origin as a covariate. Statistical analyses were conducted in R (version 4.4.2) using the Mplus Automator package (version, 1.1.1) and Mplus 8.11 (Muthén & Muthén, 2024).

## Results

Participants (*N* = 69,924) are from studies in Australia, Denmark, Sweden, and the United States that are members of the IGEMS consortium (Finkel et al., 2025a; Pedersen et al., 2013; Pedersen et al., 2019). Participants were, on average, 60 years old (range 22–103) when they completed the assessment of depressive symptoms (see Table 1 for descriptive statistics). The calendar year in which they were 10 years old ranged from 1903 to 1989. Most participants completed a high school education. Approximately half of the participants were women. Women, compared to men, reported significantly more depressive symptoms, and were less likely to come from the United States. Income inequality decreased between 1900 and ~1975 for most countries, with varying amounts of uptick in subsequent years (Supplementary Figure S3, Panel A) while GDP increased over this period (Supplementary Figure S3, Panel B).

**Table 1.** Sample descriptive statistics and comparisons between participants by sex (*N* = 69,924) Table 1. long description.

	Full sample (*N* = 69,924)	Women (*N* = 35,511)	Men (*N* = 34,413)	
Variable	Mean (SD)/% (*N*)	Mean (SD)/% (*N*)	Mean (SD)/% (*N*)	*p*
Age at assessment	60.33 (11.34)	59.92 (11.59)	60.75 (11.05)	<0.001
Zygosity % (*n*)				<0.001
DZ^a^	40% (28,282)	40% (14,213)	41% (14,069)	
MZ^b^	30% (20,876)	29% (10,372)	31% (10,504)	
OS^c^	30% (20,766)	31% (10,926)	29% (9,840)	
Country of origin				<0.001
Australia	4% (2,887)	6% (1,998)	3% (889)	
Denmark	23% (15,938)	24% (8,608)	21% (7,330)	
Sweden	63% (43,813)	66% (23,524)	59% (20,289)	
United States	10% (7,286)	4% (1,381)	17% (5,905)	
Depressive symptoms^d^	21.13 (4.41)	21.60 (4.75)	20.64 (3.98)	<0.001
Square root of depressive symptoms	4.57 (0.45)	4.62 (0.48)	4.52 (0.41)	<.0001
Calendar year at age 10	1949 (13)	1950 (14)	1949 (13)	<0.001
Calendar year at assessment	2000 (5)	2000 (5)	2000 (4)	<0.001
Top 1% index at age 10	9.75 (3.54)	9.44 (3.27)	10.08 (3.77)	<0.001
Average Top 1% index ages 5–15	9.75 (3.39)	9.44 (3.11)	10.07 (3.63)	<0.001
GDP^e^ at age 10	12.71 (4.52)	12.73 (4.65)	12.68 (4.39)	0.180
Educational attainment (ISCED^f^)	3.18 (1.86)	3.05 (1.81)	3.32 (1.90)	<0.001

a DZ, same sex dizygotic twin.

b MZ, same sex monozygotic twin.

c OS, opposite sex dizygotic twin.

d Depressive symptoms = harmonized depressive symptoms score in Cambridge Mental Disorders of the Elderly Examination (CAMDEX) units.

e GDP, Gross Domestic Product Per Capita (per thousands) in 2011 USA dollars.

f ISCED, International Standard Classification of Education level.

Genotyping information was not available for all participants. In particular, no women from the United States had genotype data. Compared to the full sample, participants with genotype data were about 3.3 years older when assessed for depressive symptoms, had lower representation from Sweden, had higher income inequality and lower GDP, and were 10 years old about 4 years earlier (Supplementary Table S1). However, participants with genotype data were comparable to the full sample with respect to depressive symptoms and educational attainment.

### Relationship of childhood income inequality to adult depressive symptoms

**Figure 1.**
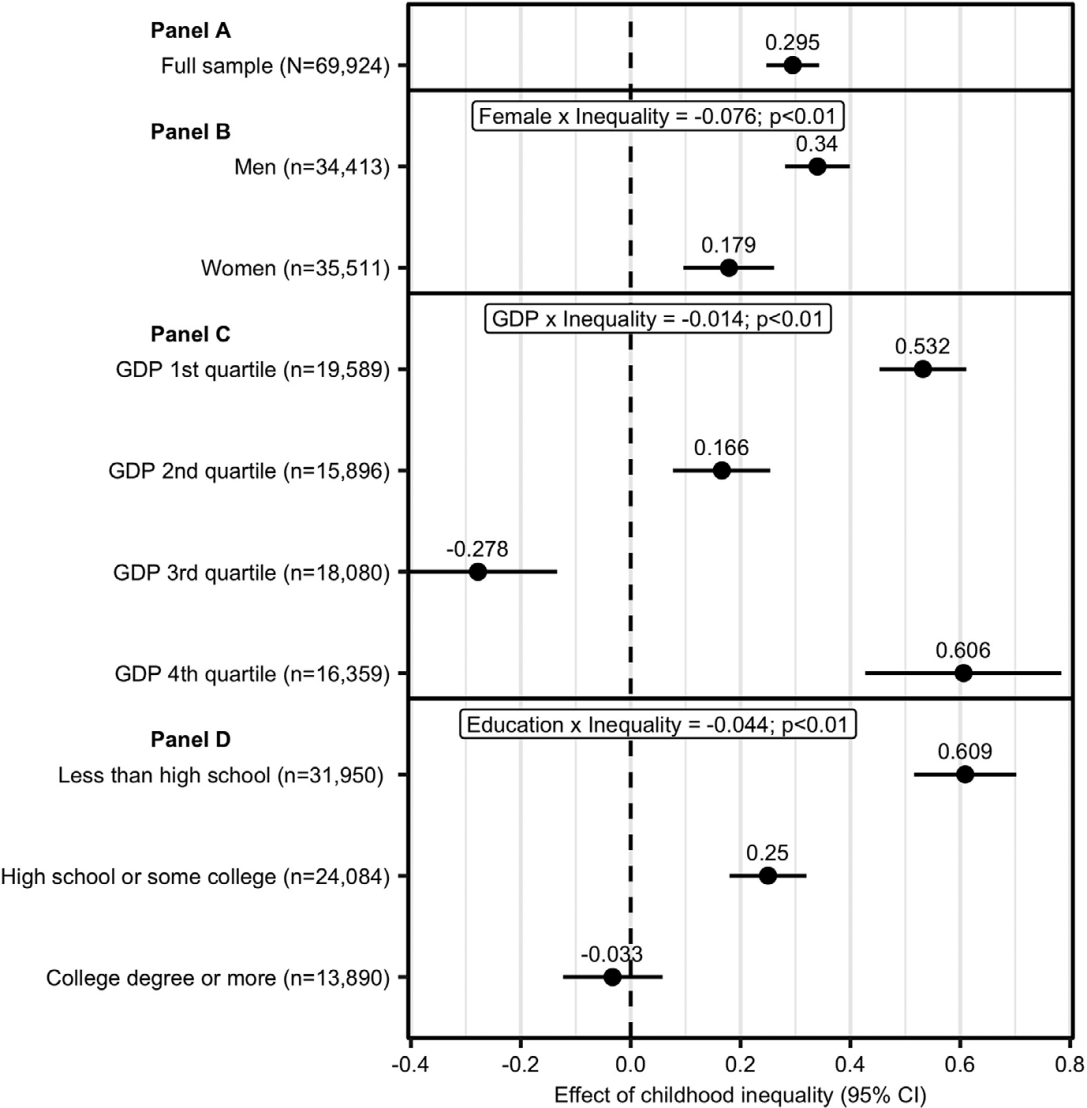
Plot of the effect of childhood inequality (95% confidence interval) on depressive symptoms for the full sample (Panel a), stratified by sex (Panel b), childhood gross domestic product (Panel c), and attained education (Panel d). The interaction parameter estimate provided in each panel inset represents each model’s continuous moderator by childhood inequality interaction term, supporting stratified analyses. Figure 1. long description.

Exposure to higher income inequality during childhood was correlated with higher depressive symptom scores. After adjusting for age, education, and sex, each 1% rise in income inequality was associated with an ~.295 higher T-score standardized depressive symptom score (*β* = .295; 95% confidence interval [CI] = .25, .34; *p* < .001) (Figure 1 Panel a).

The observed adverse effect of higher income inequality on depressive symptoms substantially differed by sex, childhood GDP, and attained education. The effect was significantly greater (Sex × Inequality interaction = −.076; *p* = .004) for men (*β* = .34; 95% CI = .28, .40; *p* < .001) compared to women (*β* = .18; 95% CI = .10, .25; *p* < .001) (Figure 1 Panel b). The adverse effect of income inequality was also modified by the country’s overall wealth (Inequality × GDP = −.018; *p* < .01) (Figure 1 Panel c). Specifically, the adverse effect of inequality on depressive symptoms was of the largest magnitude in individuals who grew up in the lowest (GDP ≤ 9,237) or highest (GDP > 15,625) GDP quantiles compared to those in the second or third quantiles. Attained education also moderated the adverse effect of inequality on depressive symptoms (Inequality × Education = −.044; *p* < .01), with the adverse effect of income inequality being the largest in individuals with less than a high school education (*β* = .609; 95% CI = .52, .70; *p* < .001) (Figure 1 Panel d). In contrast, the adverse effect of childhood income inequality on depressive symptoms was attenuated and not significant in individuals who obtained a college degree or more (*β* = −.033; 95% CI = −.12, .04; *p* = .479).

### Polygenic score analyses

Initial analyses tested the main effects of the major depressive disorder polygenic index (MDD PGI) and childhood income inequality on adult depressive symptoms, adjusting for piecewise age terms and accounting for pair dependency. Childhood inequality and GDP were correlated −0.628 in men and −0.810 in women. Given the absence of US women from the sample and the strong overlap between inequality and GDP in women, we conducted analyses within sex. Full modeling results are in Supplementary Table S2.

**Figure 2.**
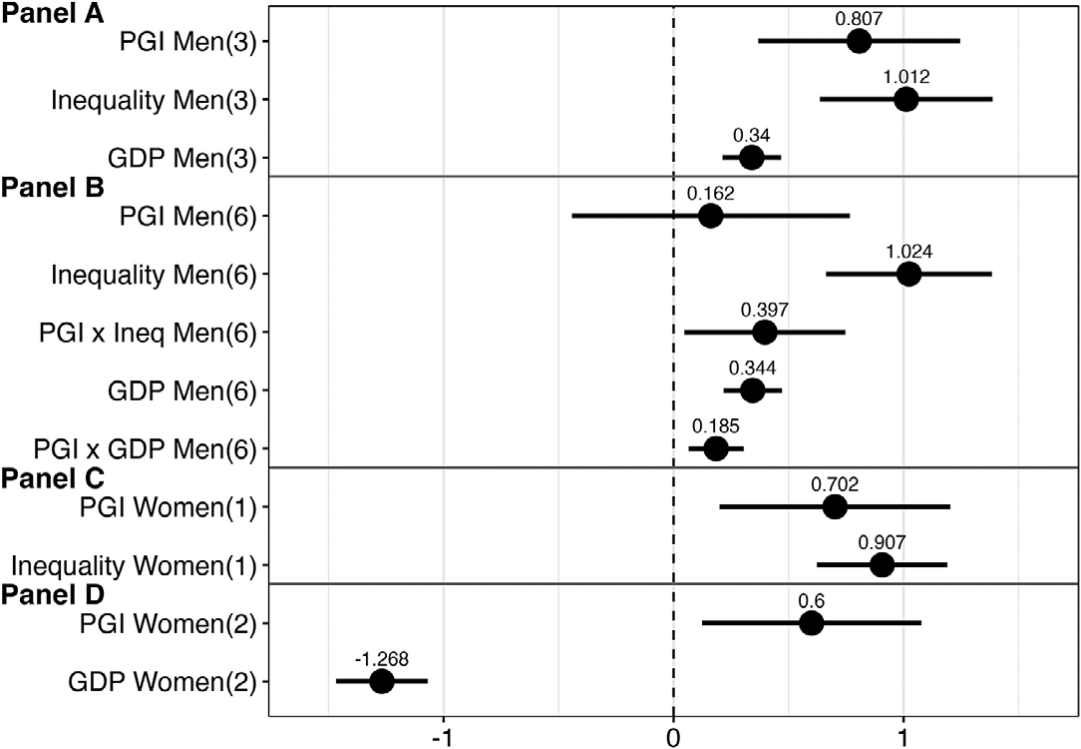
Plot of the effect of MDD PGI (major depressive disorder polygenic index), childhood inequality, and GDP (Gross Domestic Product) on depressive symptoms (with 95% confidence intervals) for men (Panels a and b) and women (Panels c and d). The figures in parentheses correspond to the models in Supplementary Table S2. For men, the main effects in Panel A are from a main effects model with all three predictors (Model 3, Supplementary Table S2). The best-fitting model is Model 6. For women, the main effects are from separate models (Models 1 and 2, Supplementary Table S2). Figure 2. long description.

In men, income inequality (*B* = 1.012; SE = 0.192; 95% CI = [0.636, 1.387]; *p* = .000), GDP (*B* = 0.340; SE = 0.065; 95% CI = [0.213, 0.467]; *p* = .000), and MDD PGI were significant main effects (*B* = 0.807; SE = 0.224; 95% CI = [0.368, 1.246]; *p* = .000) (Model 3, Supplementary Table S2), suggesting that higher childhood income inequality, GDP, and MDD PGI each contributed to higher phenotypic depressive symptoms. (The positive association between GDP and depressive symptoms may represent a statistical enhancement effect, as the Pearson correlation between GDP and depressive symptoms is .02). A further analysis evaluated PGI and childhood inequality moderation with two-way interactions of the PGI with income inequality and the PGI with childhood GDP (Model 6, Supplementary Table S2). Model fit was improved (Adjusted MLR Chi-Square Difference test [2] = 10.4802, *p* < .0053) (Figure 2, Panels a and b).

In women, income inequality (*B* = 0.907; SE = 0.145; 95% CI = [0.623, 1.190]; *p* = .000) or GDP (*B* = −1.268; SE= 0.101; 95% CI = [−1.467, −1.069]; *p* = .000), were individually significant alongside MDD PGI (*B* = 0.600–0.702; SE = 0.243–0.256; 95% CI = [0.200, 1.203] and CI = [0.124, 1.077]; *p* = 0.000–0.014) (Models 1 and 2, Supplementary Table S2; Figure 2, Panels c and d). Both the interaction of childhood income inequality (Model 4, Supplementary Table S2) and GDP with MDD PGI (Model 5, Supplementary Table S2) were nonsignificant (both MLR Chi-Square Difference test [1] *p* ≥ .7595).

Overall, results support that childhood income inequality moderates genetic influences for men, but only the main effects of both MDD PGI and childhood inequality (or GDP) are salient for women.

### Income inequality and heritability

Twin correlations (Supplementary Table S3) suggest both genetic and nonshared environmental influences on depressive symptoms. Univariate biometric models support this interpretation with significant additive genetic (unstandardized additive genetic variance = 32.08; 95% CI = 30.43; 33.73; *p* < .001) and nonshared environmental contributions (unstandardized nonshared environmental variance = 65.41; 95% CI = 63.84; 66.98; *p* < .001) to depressive symptoms. The shared environmental variance component was not significant (unstandardized common environmental variance ≤ 0.001; 95% CI = −.009; .009; *p* = .999). The heritability of depressive symptoms, averaged across all levels of income inequality, was 33% (95% CI = .31; .35; *p* < .01).

**Figure 3.**
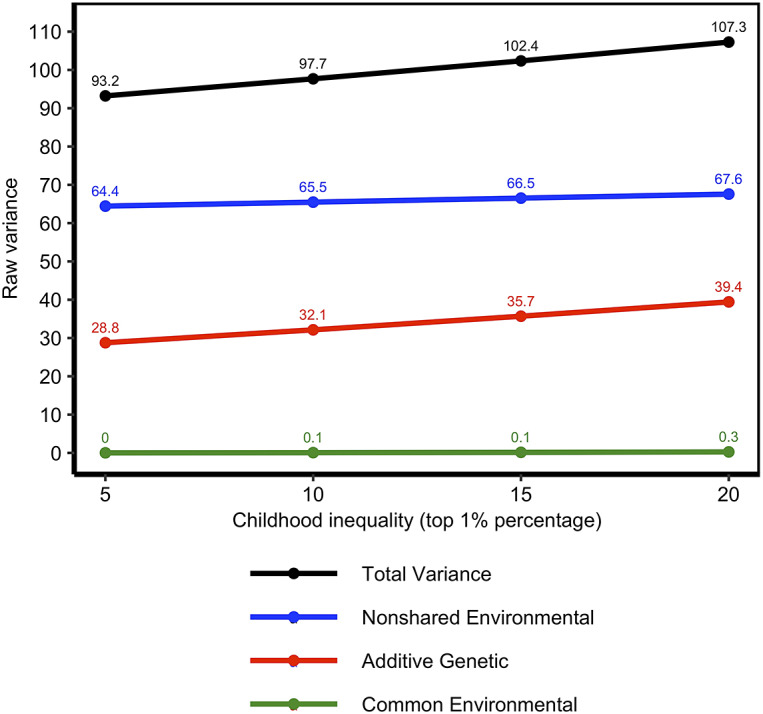
The estimated unstandardized variance components from the full gene-by-environment ACE model to examine whether childhood income inequality moderates the variance of the additive genetic (A), common environmental (C), and nonshared environmental (E) contributions to depressive symptoms. Top 1% indicates the share of the national income accounted for by the highest 1% of the population. Figure 3. long description.

Supporting our hypothesis, income inequality moderated genetic influences on depressive symptoms. In the gene-by-environment model, income inequality moderation of the additive genetic variance underlying depressive symptoms could not be omitted without significantly worse model fit (Supplementary Table S4). The additive genetic contributions to depressive symptoms accounted for a larger variance with greater income inequality (A1 parameter = .057; *p* = .016). The heritability of depressive symptoms was 30.8% at the lowest level of childhood inequality and 36.7% at the highest level of childhood inequality (Figure 3).

### Sensitivity analysis

Pooling twin studies from different birth cohorts and multiple countries addressed confounding between year and exposure to inequality, but does not account for all the unmeasured ways in which countries might differ. Sensitivity analyses were conducted where we estimated the effect of income inequality on depressive symptoms while adjusting for the effect of country. After adjusting for country, the effect of income inequality remained significantly associated with depressive symptoms (*β* = .308; 95% CI = .233, .382; *p* < .001).

## Discussion

Our study addresses three questions regarding the relationship between country-level income inequality in childhood and depressive symptoms in midlife or older adulthood. Our first question concerned the main effect of childhood exposure to income inequality on adult depressive symptoms. We confirmed that higher levels of income inequality in childhood were associated with higher depressive symptom scores in adulthood, adjusting for covariates. In general, effects were stronger for men than for women, for those who grew up at a time when their country’s GDP was either low or very high, and for those who attained less education. A previous meta-analysis, in contrast, found that associations between inequality and depression were more likely in women than in men, although often there was no test for a gender effect (Patel et al., 2018). These authors suggest that elevated depression may reflect adolescent girls being particularly sensitive to social status (Patel et al., 2018). We would also suggest that young men may be particularly vulnerable to worry about how inequality can affect their job opportunities, with that anxiety providing a mechanism to explain the association between inequality and depression (Layte et al., 2019; Rakesh et al., 2025a).

The nonlinear moderation by GDP shows how inequality and GDP may work together. We found that the most depressogenic conditions are where there is both high inequality and high GDP (e.g. a situation of extreme wealth concentration) or where there is both high inequality and low GDP (e.g. a situation of widespread concentrated poverty). In our study, the best example of the former may be the United States in the most recent years included in the analyses. The best example of the latter may be Sweden at the beginning of WWI, although GDP in Sweden was then moderate rather than low by global standards. Historically, more extreme examples might be the United States during the Gilded Age and Ireland during the Great Famine (Piketty, 2014).

Prior research had suggested that associations between inequality and depression may be greater when the country is less wealthy, but also that the mechanisms explaining associations between inequality and depression may differ between higher- and lower-income countries (Rakesh et al., 2025a). For instance, effects of income inequality on social capital and societal norms may be more salient in explaining associations between inequality and depression in the context of higher country-level wealth (Rakesh et al., 2025a).

Our results add to the literature on childhood and adolescent adverse exposures and adult mental health. The negative effects of childhood income inequality on adult depressive symptoms were roughly equal to the negative effect of lower education (per 1 ISCED unit) on depressive symptoms (β = −.252; 95% Confidence Interval (CI) = −.29, −.21; p<.001). Importantly, as operationalized in this study, income inequality represents an exogenous indicator of exposure to a potentially relevant social determinant of health.

Our second and third questions concerned the role of gene-by-environment interactions in shaping depressive symptoms. While exogenous indicators of inequality are not unusual in studies of income inequality in individuals in a society, they are uncommon in genetically informed studies. Exogenous measures of the environment are important in GxE work because of possible genetic confounds when self- or parent-reported stresses are the environmental factor.

Using a PGI, we asked whether individuals at greater genetic risk for depression were more sensitive to childhood inequality, following a diathesis-stress model. This prediction was supported for men, while for women, the MDD PGI and childhood inequality were each predictive of adult depressive symptoms, with no gene-by-environment interaction. The results for women are consistent with a report from the Health and Retirement Study, where polygenic scores and self-reported recent stressful life events predicted depressive symptoms additively (Musliner et al., 2015). The results for men fit a diathesis-stress model. Based on both regressions of inequality on depressive symptoms and GxE analyses using polygenic scores, this raises the question of whether men may be more sensitive to childhood inequality than women.

Using a twin design that decomposes variance into additive genetic, shared environment, and nonshared environmental components, we asked whether exposure to inequality in childhood moderated additive genetic influences on depressive symptoms in adulthood. Gene-by-environment interaction would be evidenced if the strength of genetic influence was moderated by environmental circumstance (Boardman et al., 2013; Reiss et al., 2013). Results supported this conclusion, with the heritability of depressive symptoms lower at the lowest level of childhood inequality and higher at the highest level of inequality, consistent with the prediction that heritability for an unfavorable health condition will be lower in advantaged environments and maximized in adverse environments. The results do not tell us how inequality stimulates depression. The results do tell us that severe inequality has ramifications for individual differences in depressive symptoms through moderating genetic predispositions (a process described by Selita & Kovas, 2019). With greater equality, genetic predispositions for depression may be suppressed, along with implications of depression for subsequent social and health outcomes.

Strengths of the study include having access to representative samples from multiple countries and a wide range of birth years, such that the results are not tied to a particular country or birth cohort. On the other hand, generalizability is limited, as all the countries included in our analysis are high-income countries – although this was not true for the earliest-born Swedish and Danish birth cohorts – and Scandinavian countries have less inequality than many countries in the world. Additionally, the included studies lack racial and ethnic diversity. There is insufficient inclusion of participants other than non-Hispanic White across birth cohorts to perform stratified analyses. While the Top 1% index offers the advantage of providing an exogenous measure of exposure, by definition, it is a blunt indicator as it presumes similar exposure for everyone in the country. A further presumption is that the same Top 1% score represents a similar exposure in different decades, for example, 1920 and 1970. However, observed results may partly reflect unmeasured country-period factors that are correlated with inequality rather than inequality itself.

Use of the Top 1% index for income inequality is perhaps not ideal. The Gini coefficient is the most widely used measure of income inequality due to its easy interpretability and its use of the entire income distribution. However, Gini scores are not available historically for many countries due to a lack of administrative data from which it is calculated. Top 1% is available for a much longer historical time period and is correlated with broader distributional measures, including the Gini coefficient (Kawachi & Kennedy, 1997), although this correlation may be stronger for measures of mid-distribution inequality than for the extreme top of the income distribution. Given the age of our respondents and the inequality conditions within their countries at the time of key developmental periods, the use of Top 1% data was required to provide the necessary data extending back into the early twentieth century.

Finally, we lack the information from participants that would be required to probe mechanisms for the observed associations at either a community or individual level. Mechanisms to explore include poverty, financial strain (Finkel et al., 2025b), sense of unfairness, reduced public goods (Rakesh et al., 2025a), frayed norms for collective action (Rakesh et al., 2025a), and constrained opportunities for one’s future.

## Supporting information

10.1017/S0033291726104620.sm001 Petkus et al. supplementary material Petkus et al. supplementary material

## Data Availability

This research used third-party data and materials accessed through the IGEMS consortium. The following is a list of participating studies with links to how to access or to request data from each study: SATSA data are publicly available through the National Archive of Computerized Data on Aging (NACDA). Pedersen, Nancy L. Swedish Adoption/Twin Study on Aging (SATSA), 1984, 1987, 1990, 1993, 2004, 2007, and 2010. Inter-university Consortium for Political and Social Research [distributor], 2015-05-13. https://doi.org/10.3886/ICPSR03843.v2 Investigators may apply for OCTO-Twin data through the University of Gothenburg. See https://www.gu.se/en/research/the-octo-twin-study-origins-of-variance-in-the-old-old Investigators may apply for Gender data through the National E-infrastructure for Aging Research (NEAR). See https://www.near-aging.se/studies-included/application/ SALT data are hosted by the Swedish Twin Registry. https://ki.se/en/research/research-infrastructure-and-environments/core-facilities-for-research/the-swedish-twin-registry For access to data from the Danish Twin Registry, see https://www.sdu.dk/en/om_sdu/institutter_centre/ist_sundhedstjenesteforsk/centre/dtr/researcher The consent forms used in the original MTSADA do not allow for placing that data in a public-access repository. Researchers interested in working with deidentified MTSADA data should contact the Minnesota Center for Twin and Family Research at mctfrdm@umn.edu For VETSA data, see instructions to researchers: https://psychiatry.ucsd.edu/research/programs-centers/vetsa/researchers.html All MIDUS data are archived and made publicly available – and are thus shareable – via the University of Michigan Inter-university Consortium of Political and Social Research (ICPSR) https://www.icpsr.umich.edu/web/ICPSR/series/203. Brim, Orville Gilbert, Baltes, Paul B., Bumpass, Larry L., Cleary, Paul D., Featherman, David L., Hazzard, William R., … Shweder, Richard A. Midlife in the United States (MIDUS 1), 1995-1996. Inter-university Consortium for Political and Social Research [distributor], 2020-09-28. https://doi.org/10.3886/ICPSR02760.v19.
Ryff, Carol D., Almeida, David M., Ayanian, John Z., Carr, Deborah S., Cleary, Paul D., Coe, Christopher, … Williams, David R. Midlife in the United States (MIDUS 2), 2004-2006. Inter-university Consortium for Political and Social Research [distributor], 2021-09-15. https://doi.org/10.3886/ICPSR04652.v8
Ryff, Carol D., Seeman, Teresa, and Weinstein, Maxine. Midlife in the United States (MIDUS 2): Biomarker Project, 2004-2009. Inter-university Consortium for Political and Social Research [distributor], 2025-06-18. https://doi.org/10.3886/ICPSR29282.v11
Ryff, Carol, Almeida, David, Ayanian, John, Binkley, Neil, Carr, Deborah S., Coe, Christopher, … Williams, David. Midlife in the United States (MIDUS 3), 2013-2014. Inter-university Consortium for Political and Social Research [distributor], 2019-04-30. https://doi.org/10.3886/ICPSR36346.v7 NAS-NRC Twin Registry data are publicly available through the National Archive of Computerized Data on Aging (NACDA). Gatz, Margaret, and Butler, David (David Alan). National Academy of Sciences-National Research Council Twin Registry (NAS-NRC Twin Registry), 1958-2013 [RESTRICTED]. Inter-university Consortium for Political and Social Research [distributor], 2020-11-16. https://doi.org/10.3886/ICPSR36234.v6 Individual-level data from OATS cannot be made publicly available due to ethical and legal restrictions. However, access to this data may be provided subject to approval of the OATS Governance Committee. Application for access to OATS data can be made via Dementias Platform Australia (DPAU): https://www.dementiasplatform.com.au/ Access to A50s data is restricted due to the ethical guidelines governing the study, but may be accessible following ethical review and data transfer agreements. Please contact Nicholas Martin (nick.martin@qimrberghofer.edu.au) with queries related to accessing A50s data. Code used in data analyses is available on the IGEMS website https://dornsife.usc.edu/cesr/IGEMS/

## Supplementary Material

The supplementary material for this article can be found at http://doi.org/10.1017/S0033291726104620.

## Table 1. Long description

Beginning at the top, the table lists variables in the first column: age at assessment, zygosity, country of origin, depressive symptoms, square root of depressive symptoms, calendar year at age 10, calendar year at assessment, Top 1 percent index at age 10, average Top 1 percent index ages 5 to 15, G D P at age 10, and educational attainment (I S C E D). For each variable, the next columns provide values for the full sample, women, and men, with sample sizes specified. For age at assessment, the mean is 60.33 years (standard deviation 11.34) for the full sample, 59.92 (11.59) for women, and 60.75 (11.05) for men, with a p-value less than 0.001. Zygosity is divided into D Z (same sex dizygotic twin), M Z (same sex monozygotic twin), and O S (opposite sex dizygotic twin), with respective percentages and counts for each group. Country of origin is distributed as Australia (4 percent full sample, 6 percent women, 3 percent men), Denmark (23 percent, 24 percent, 21 percent), Sweden (63 percent, 66 percent, 59 percent), and United States (10 percent, 4 percent, 17 percent). Depressive symptoms are reported as mean scores in C A M D E X units: 21.13 (4.41) full sample, 21.60 (4.75) women, 20.64 (3.98) men, p-value less than 0.001. The square root of depressive symptoms, calendar years, Top 1 percent indices, G D P at age 10, and educational attainment (I S C E D) are similarly detailed, with all values and p-values provided. Footnotes clarify abbreviations: D Z, M Z, O S, C A M D E X, G D P, and I S C E D.

Navigate back to Table 1..

## Figure 1. Long description

Panel A at the top shows the full sample (N equals 69,924) with a positive effect estimate of 0.295 and confidence interval crossing zero. Panel B splits by sex: men (n equals 34,413) have an estimate of 0.34, women (n equals 35,511) have 0.179, with a significant negative interaction for female by inequality of minus 0.076, p less than 0.01. Panel C stratifies by childhood G D P quartile: first quartile (n equals 19,589) is not shown, second (n equals 15,896) is 0.166, third (n equals 18,080) is minus 0.278, fourth (n equals 16,359) is 0.532 and 0.606, with a significant G D P by inequality interaction of minus 0.014, p less than 0.01. Panel D stratifies by education: less than high school (n equals 31,950) is 0.609, high school or some college (n equals 24,084) is 0.25, college degree or more (n equals 13,890) is minus 0.033, with a significant education by inequality interaction of minus 0.044, p less than 0.01. All estimates are plotted as points with horizontal lines for 95 percent confidence intervals along the x axis labeled effect of childhood inequality.

Navigate back to Figure 1..

## Figure 2. Long description

There are four horizontal panels labeled Panel A, Panel B, Panel C, and Panel D, arranged vertically. The x-axis represents effect size, ranging from approximately -1.5 to 1.5, with a dashed vertical line at zero. The y-axis lists predictors for each panel. Panel A (top) shows main effects for men: P G I Men (3) at 0.807, Inequality Men (3) at 1.012, and G D P Men (3) at 0.34, each with horizontal lines indicating 95 percent confidence intervals. Panel B (second) shows the best-fitting model for men: P G I Men (6) at 0.162, Inequality Men (6) at 1.024, P G I x Inequality Men (6) at 0.397, G D P Men (6) at 0.344, and P G I x G D P Men (6) at 0.185, each with confidence intervals. Panel C (third) shows main effects for women: P G I Women (1) at 0.702 and Inequality Women (1) at 0.907, both with confidence intervals. Panel D (bottom) shows P G I Women (2) at -1.268 and G D P Women (2) at 0.6, both with confidence intervals. All effect sizes are marked by black dots, with horizontal lines spanning their confidence intervals. Positive effects are mostly observed except for P G I Women (2), which is negative.

Navigate back to Figure 2..

## Figure 3. Long description

The x axis shows childhood inequality as the top 1 percent percentage, ranging from 5 to 20. The y axis shows raw variance from 0 to 110. Four lines are plotted: total variance (black), nonshared environmental (blue), additive genetic (red), and common environmental (green). At each x axis value (5, 10, 15, 20), the black line rises from 93.2 to 107.3, the blue line rises from 64.4 to 67.6, the red line rises from 28.8 to 39.4, and the green line remains near zero (0 to 0.3). The legend at the bottom identifies each line by color and label. The graph shows that as childhood inequality increases, total and additive genetic variance increase, nonshared environmental variance increases slightly, and common environmental variance remains minimal.

Navigate back to Figure 3..

## References

[r1] Bleys, D., Luyten, P., Soenens, B., & Claes, S. (2018). Gene-environment interactions between stress and 5-HTTLPR in depression: A meta-analytic update. Journal of Affective Disorders, 226, 339–345. 10.1016/j.jad.2017.09.050 29031184

[r2] Boardman, J. D., Daw, J., & Freese, J. (2013). Defining the environment in gene-environment research: Lessons from social epidemiology. American Journal of Public Health, 103 (Suppl 1), S64–S72. 10.2105/AJPH.2013.301355 23927514 PMC3786759

[r3] Caspi, A., Sugden, K., Moffitt, T. E., Taylor, A., Craig, I. W., Harrington, H., … Poulton, R. (2003). Influence of life stress on depression: Moderation by a polymorphism in the 5-HTT gene. Science, 301(5631), 386–389. 10.1126/science.1083968 12869766

[r4] Chang, C. C., Chow, C. C., Tellier, L. C., Vattikuti, S., Purcell, S. M., & Lee, J. J. (2015). Second-generation PLINK: Rising to the challenge of larger and richer datasets. GigaScience, 4, 7. 10.1186/s13742-015-0047-8 25722852 PMC4342193

[r5] Colodro-Conde, L., Rijsdijk, F., Tornero-Gómez, M. J., Sánchez-Romera, J. F., & Ordoñana, J. R. (2015). Equality in educational policy and the heritability of educational attainment. PLoS One, 10(11), e0143796. 10.1371/journal.pone.0143796 26618539 PMC4664401

[r6] Daníelsdóttir, H. B., Aspelund, T., Shen, Q., Halldorsdottir, T., Jakobsdóttir, J., Song, H., … Valdimarsdóttir, U. A. (2024). Adverse childhood experiences and adult mental health outcomes. JAMA Psychiatry, 81(6), 586–594. 10.1001/jamapsychiatry.2024.0039 38446452 PMC10918580

[r7] Duncan, L. E., & Keller, M. C. (2011). A critical review of the first 10 years of candidate gene-by-environment interaction research in psychiatry. The American Journal of Psychiatry, 168(10), 1041–1049. 10.1176/appi.ajp.2011.11020191 21890791 PMC3222234

[r8] Duncan, G., & Murnane, R. (2011). The American dream, then and now. In G. Duncan & R. Murnane (Eds.), Whither opportunity? Rising inequality, schools, and children’s life chances (pp. 2–24). Russell Sage Foundation. https://doi.org/stable/10.7758/9781610447515

[r9] Finch, B. K., Nimmagadda, S., Finkel, D., Gatz, M., Reynolds, C. A., Nygaard, M., Catts, V., Thalamuthu, A., Sachdev, P., Ericsson, M., Karlsson, I., Thorvaldsson, V., & Hassing, L. (2026). Moderating effects of educational inequality on education polygenic scores, attained education and dementia-risk relationships. Social Science & Medicine: Population Health, 34, 101914. 10.1016/j.ssmph.2026.101914 PMC13089079 42005573

[r10] Finkel, D., Finch, B. K., Gatz, M., Christensen, K., Franz, C. E., Karlsson, I. K., … IGEMS Consortium. (2025a). The interplay of genes and environment across multiple studies (IGEMS) consortium after fifteen years. Twin Research and Human Genetics: The Official Journal of the International Society for Twin Studies, 1–10. 10.1017/thg.2025.10036 PMC13033607 41416461

[r11] Finkel, D., Hyde, M., Hasselgren, C., Sacco, L., Sindi, S., & Nilsen, C. (2025b). Both childhood and adult perceived financial strain impact age trajectories of change in emotional health in late adulthood. Aging & Mental Health, 29(8), 1497–1504. 10.1080/13607863.2025.2464709 39945660 PMC12213204

[r12] Gatz, M., Reynolds, C. A., Finkel, D., Hahn, C. J., Zhou, Y., & Zavala, C. (2015). Data harmonization in aging research: Not so fast. Experimental Aging Research, 41(5), 475–495. 10.1080/0361073X.2015.1085748 26524232 PMC4772674

[r13] Hasell, J., & Arriagada, P. (2023). *OWID data collection: Inequality and poverty.* Published online at OurWorldinData.org. https://ourworldindata.org/owid-data-collection-inequality-and-poverty.

[r14] Howard, D. M., Adams, M. J., Clarke, T. K., Hafferty, J. D., Gibson, J., Shirali, M., … McIntosh, A. M. (2019). Genome-wide meta-analysis of depression identifies 102 independent variants and highlights the importance of the prefrontal brain regions. Nature Neuroscience, 22(3), 343–352. 10.1038/s41593-018-0326-7. 30718901 PMC6522363

[r15] Hoynes, H., Schanzenbach, D. W., & Almond, D. (2016). Long-run impacts of childhood access to the safety net. The American Economic Review, 106(4), 903–934.

[r16] IGEMS Consortium. (2022). *IGEMS/PGS_pipeline.* GitHub. https://github.com/IGEMS/PGS_pipeline

[r17] Illius, S., Eder, J., Vogel, S., & Alexander, N. (2026). The predictive value of polygenic risk scores for depression in gene-environment interaction studies: A systematic review. Translational Psychiatry, 16, 121. 10.1038/s41398-025-03793-7 41741414 PMC12960946

[r18] International HapMap 3 Consortium, Altshuler, D. M., Gibbs, R. A., Peltonen, L., Altshuler, D. M., Gibbs, R. A., … McEwen, J. E. (2010). Integrating common and rare genetic variation in diverse human populations. Nature, 467(7311), 52–58. 10.1038/nature09298 20811451 PMC3173859

[r19] Johnson, W., Kyvik, K. O., Skytthe, A., Deary, I. J., & Sørensen, T. I. (2011). Education modifies genetic and environmental influences on BMI. PLoS One, 6(1), e16290. 10.1371/journal.pone.0016290 21283825 PMC3023797

[r20] Kawachi, I., & Kennedy, B. P. (1997). The relationship of income inequality to mortality: Does the choice of indicator matter? Social Science and Medicine, 45(7), 1121–1127. 10.1016/s0277-9536(97)00044-0 9257403

[r21] Kendler, K. S., Gatz, M., Gardner, C. O., & Pedersen, N. L. (2006). A Swedish national twin study of lifetime major depression. The American Journal of Psychiatry, 163(1), 109–114. 10.1176/appi.ajp.163.1.109 16390897

[r22] Kenworthy, L. (2016). *Is Income inequality harmful?* https://lanekenworthy.net/is-income-inequality-harmful/

[r23] Kirkbride, J. B., Anglin, D. M., Colman, I., Dykxhoorn, J., Jones, P. B., Patalay, P., … Griffiths, S. L. (2024). The social determinants of mental health and disorder: Evidence, prevention and recommendations. World Psychiatry: Official Journal of the World Psychiatric Association (WPA), 23(1), 58–90. 10.1002/wps.21160 38214615 PMC10786006

[r24] Layte, R., McCrory, C., Cheallaigh, C. N., Bourke, N., Kivimaki, M., Ribeiro, A. I., … Vineis, P. (2019). A comparative analysis of the status anxiety hypothesis of socio-economic inequalities in health based on 18,349 individuals in four countries and five cohort studies. Scientific Reports, 9(1), 796. 10.1038/s41598-018-37440-7 30692559 PMC6349896

[r25] Lloyd-Jones, L. R., Zeng, J., Sidorenko, J., Yengo, L., Moser, G., Kemper, K. E., … Visscher, P. M. (2019). Improved polygenic prediction by Bayesian multiple regression on summary statistics. Nature Communications, 10(1), 5086. 10.1038/s41467-019-12653-0 PMC6841727 31704910

[r26] Lynch, J. W., Kaplan, G. A., Pamuk, E. R., Cohen, R. D., Heck, K. E., Balfour, J. L., & Yen, I. H. (1998). Income inequality and mortality in metropolitan areas of the United States. American Journal of Public Health, 88(7), 1074–1080. 10.2105/ajph.88.7.1074 9663157 PMC1508263

[r27] McGue, M., & Christensen, K. (2003). The heritability of depression symptoms in elderly Danish twins: Occasion-specific versus general effects. Behavior Genetics, 33(2), 83–93. 10.1023/a:1022545600034 14574144

[r28] Messias, E., Eaton, W. W., & Grooms, A. N. (2011). Economic grand rounds: Income inequality and depression prevalence across the United States: An ecological study. Psychiatric Services (Washington, D.C.), 62(7), 710–712. 10.1176/ps.62.7.pss6207_0710 21724781

[r29] Musliner, K. L., Seifuddin, F., Judy, J. A., Pirooznia, M., Goes, F. S., & Zandi, P. P. (2015). Polygenic risk, stressful life events and depressive symptoms in older adults: A polygenic score analysis. Psychological Medicine, 45(8), 1709–1720. 10.1017/S0033291714002839 25488392 PMC4412793

[r30] Muthén, L. K., & Muthén, B. O. (2024) *Mplus Version 8.11.* https://www.statmodel.com/.

[r31] Patel, V., Burns, J. K., Dhingra, M., Tarver, L., Kohrt, B. A., & Lund, C. (2018). Income inequality and depression: A systematic review and meta-analysis of the association and a scoping review of mechanisms. World Psychiatry: Official Journal of the World Psychiatric Association (WPA), 17(1), 76–89. 10.1002/wps.20492 29352539 PMC5775138

[r32] Pedersen, N. L., Christensen, K., Dahl, A. K., Finkel, D., Franz, C. E., Gatz, M., … Reynolds, C. A. (2013). IGEMS: The consortium on interplay of genes and environment across multiple studies. Twin Research and Human Genetics: The Official Journal of the International Society for Twin Studies, 16(1), 481–489. 10.1017/thg.2012.110 23186995 PMC3699700

[r33] Pedersen, N. L., Gatz, M., Finch, B. K., Finkel, D., Butler, D. A., Dahl Aslan, A., … Whitfield, K. E. (2019). IGEMS: The consortium on interplay of genes and environment across multiple studies – an update. Twin Research and Human Genetics: The Official Journal of the International Society for Twin Studies, 22(6), 809–816. 10.1017/thg.2019.76 31544729 PMC7056501

[r34] Pickett, K. E., & Wilkinson, R. G. (2015). Income inequality and health: A causal review. Social Science & Medicine (1982), 128, 316–326. 10.1016/j.socscimed.2014.12.031 25577953

[r35] Piketty, T. (2014). Capital in the twenty-first century. Harvard University Press. 10.1111/1468-4446.12115 25516350

[r36] Purcell, S., Neale, B., Todd-Brown, K., Thomas, L., Ferreira, M. A., Bender, D., … Sham, P. C. (2007). PLINK: A tool set for whole-genome association and population-based linkage analyses. American Journal of Human Genetics, 81(3), 559–575. 10.1086/519795 17701901 PMC1950838

[r37] Rakesh, D., Shiba, K., Lamont, M., Lund, C., Pickett, K. E., VanderWeele, T. J., & Patel, V. (2025a). Economic inequality and mental health: Causality, mechanisms, and interventions. Annual Review of Clinical Psychology, 21(1), 353–377. 10.1146/annurev-clinpsy-081423-025710 40333273

[r38] Rakesh, D., Tsomokos, D. I., Vargas, T., Pickett, K. E., & Patel, V. (2025b). Macroeconomic income inequality, brain structure and function, and mental health. Nature Mental Health, 3, 1318–1330. 10.1038/s44220-025-00508-1

[r39] Reiss, D., Leve, L. D., & Neiderhiser, J. M. (2013). How genes and the social environment moderate each other. American Journal of Public Health, 103(Suppl 1), S111–S121. 10.2105/AJPH.2013.301408 23927504 PMC3778406

[r40] Selita, F., & Kovas, Y. (2019). Genes and Gini: What inequality means for heritability. Journal of Biosocial Science, 51(1), 18–47. 10.1017/S0021932017000645 29388530

[r41] Subramanian, S. V., & Kawachi, I. (2006). Whose health is affected by income inequality? A multilevel interaction analysis of contemporaneous and lagged effects of state income inequality on individual self-rated health in the United States. Health &Place, 12(2), 141–156. 10.1016/j.healthplace.2004.11.001 16338630

[r42] UNESCO Institute for Statistics (2012). *Data from International Standard Classification of Education (ISCED 2011).* https://uis.unesco.org/en/topic/international-standard-classification-education-isced.

