# Supplementary material for: The long reach of childhood income inequality: a multinational twin study of gene–environment interplay on adult depressive symptoms
Source: Psychol Med. 2026 Jun 8;56:e182. doi: 10.1017/S0033291726104620 (PMC13254727; doi:10.1017/S0033291726104620)
Supplement: Petkus et al. supplementary material [file S0033291726104620sup001.pdf]

Supporting Information for

The Long Reach of Childhood Income Inequality: A Multinational Twin Study of Gene-Environment Interplay on Adult Depressive Symptoms

Andrew J. Petkus[*,1], Chandra A. Reynolds[*,2], Brian K. Finch[3,4], Kyla Thomas[3], Christopher R. Beam[5,6], Vibeke S. Catts[7], Malin Ericcson[8], Deborah G. Finkel[3,9], Carol E. Franz[10], William S. Kremen[10], Lisbeth Aagaard Larsen[11], Nicholas G. Martin[12], Matt McGue[13], Miriam A. Mosing[8,14], Jenae M. Neiderhiser[15], Marianne Nygaard[11], Nancy L. Pedersen[8], Anbupalam Thalamuthu[7], Keith E. Whitfield[16], Margaret Gatz [**,3], for the IGEMS Consortium

1 Department of Neurology, University of Southern California, Los Angeles, CA 90033
2 Institute for Behavioral Genetics and Department of Psychology and Neuroscience, University of Colorado Boulder, Boulder, CO 80303
3 Center for Economic and Social Research University of Southern California, Los Angeles, CA 90089
4 Department of Sociology and Spatial Sciences, University of Southern California, Los Angeles, CA 90089
5 Department of Psychology, University of Southern California, Los Angeles, CA 90089
6 Leonard Davis School of Gerontology, University of Southern California, Los Angeles, CA 90089
7 Centre for Healthy Brain Ageing (CHeBA), Discipline of Psychiatry and Mental Health, School of Clinical Medicine, UNSW, Sydney, 2052 Australia
8 Department of Medical Epidemiology and Biostatistics, Karolinska Institutet, SE-171 77 Stockholm, Sweden
9 Institute for Gerontology, School of Health and Welfare, Jönköping University, SE-551 11 Jönköping, Sweden
10 Department of Psychiatry, University of California San Diego, La Jolla, CA 92093
11 The Danish Twin Registry, Department of Public Health, University of Southern Denmark, DK-5230 Odense, Denmark
12 Mental Health and Neuroscience, QIMR Berghofer Medical Research Institute, Brisbane, 4006 Australia
13 Department of Psychology, University of Minnesota Twin Cities, Minneapolis, MN 55455
14 Max Planck Institute for Empirical Aesthetics, 60322 Frankfurt, Germany
15 Department of Psychology, The Pennsylvania State University, University Park, PA 16802
16 Program for Research on Men's Health, Hopkins Center for Health Disparities Research, Johns Hopkins University School of Public Health, Baltimore, MD 21205

* Andrew J. Petkus and Chandra A. Reynolds contributed equally to this work

**Corresponding author:
Margaret Gatz
Email: gatz@usc.edu

Contents

- Detailed Description of Included Samples
- Harmonized Depressive Symptoms Scale
- Figure S1. Correlation matrix with distributions of the main variables of interest by sex.
- Figure S2. The gene-by-environment model to examine whether childhood inequality moderates the variance additive genetic (A), common environmental (C), and nonshared environmental (E) contributions to depressive symptoms.
- Figure S3. Plot of income inequality (Panel A) and gross domestic product (GDP; Panel B) by year.
- Figure S4. Results of MPLUS Tree analyses of the effect of childhood inequality on depressive symptoms and moderation by sex, attained education, and GDP.
- Table S1. Sample descriptive statistics comparing participants by polygenic index score availability
- Table S2. Twin correlations by monozygotic, dizygotic, and opposite-sex pairs
- Table S3. Model fit statistics from the childhood income inequality moderation of the genetic and environmental contributions to depressive symptoms.

Detailed Description of Included Samples

All samples included in the analyses are part of the Interplay of Genes and Environment in Multiple Studies (IGEMS) consortium[1,2].

Denmark. Participant data were from the first wave of two studies of the Danish Twin Registry (DTR): the Middle Age Danish Twins Study (MADT)[3] and Longitudinal Study of Aging Danish Twins (LSADT)[4,5]. MADT includes both same-sex and opposite-sex twin pairs born between 1931 and 1952, who were 45 to 68 years when first assessed in 1998. LSADT began in 1995, with a cohort-sequential design including same sex twin pairs born between 1890 and 1930, who were 70 to 96 years at the intake wave.

Sweden. Six studies are included that drew participants from the Swedish Twin Register (STR)[6], a population-based register of twins born in Sweden. The Swedish Adoption/Twin Study of Aging (SATSA), followed same-sex twins who had been reared apart and a matched sample of twins reared together, born between1895 and1935, with the first assessment of depressive symptoms conducted in 1987[7]. The Origins of Variance in the Oldest-Old (OCTO-Twin)[8] followed same-sex twin pairs born between 1893 and 1913 who were aged 80 and older at the 1991 baseline assessment. The “Ageing in Women and Men: A Longitudinal Study of Gender Differences in Health Behavior and Health among Elderly” (GENDER)[9] enrolled opposite-sex twin pairs born between 1906 and 1925 with a baseline assessment in 1995. The Twin-Offspring Study in Sweden (TOSS)[10] assessed same sex twin pairs born between 1943 and 1971 who were parents of teenagers and aged 32 to 59 years at intake assessments in 1997 and 2005. The Screening Across the Lifespan Twin Study (SALT)[6] is a study of all same- and opposite-sex twins born before 1958 with one wave of interview data collected by telephone between 1998 and 2002. SALT overlapped with SATSA, Octo-Twin and Gender participants, and only one score was included in analysis, with the earliest assessment prioritized.

Australia. Two partly overlapping Australian samples born between 1899 and 1944 were drawn from the Australian Twin Registry as well as volunteers from the community: the longitudinal Older Australian Twins Study (OATS)[11,12] that began in 2006 recruiting twins aged 65 years and older, and the Australian over 50’s study (A50)[13] with twins contacted via one wave of questionnaire between 1993-1995 and ranging in age from 50 to 94 years. Only one score was included for each individual whether they were participants in one or both studies.

United States. Participant data were from five US population-representative twin studies. The Minnesota Twin Study of Adult Development and Aging (MTSADA)[14] collected data from same-sex twin pairs born between 1897 and 1970 and aged 60 and older with a baseline assessment between 1984 and 1994. The longitudinal National Academy of Sciences-National Research Council Twin Registry (NAS-NRC)[15] followed male twin pairs born between 1917 and 1927 who served in the military, with depressive symptom data collected in 1998 when participants were aged 70 to 82. The longitudinal Vietnam Era Twin Study of Aging (VETSA)[16] consisted of male twins born between 1943 and 1954 who served in the military at some time during the Vietnam era (1965-1975) with the first assessment conducted at ages 51 to 61 years of age between 2003 through 2008. The cross-sectional Carolina African American Twin Study of Aging (CAATSA)[17,18] is a study of North Carolinian African American twins who were in their 20s through 80s when assessed between1999 to 2003. The Midlife Development in the United States (MIDUS) study includes a twin subsample born between 1920 and 1970 formed partly by snowball recruitment[19] aged 34 to 82 years and first assessed by telephone in 2004 to 2006.

References

1. Pedersen NL, Christensen K, Dahl A, Finkel D, Franz C, Gatz M, Horwitz BN, Johansson B, Johnson W, Kremen WS, Lyons MJ, Malmberg B, McGue M, Neiderhiser JM, Peterson I, Reynolds CA (2013) IGEMS: The Consortium on interplay of genes and environment across multiple studies. *Twin Research and Human Genetics, 16*, 481–489.
2. Pedersen NL, Gatz M, Finch BK, Finkel D, Butler DA, Dahl Aslan A, Franz C, Kaprio J, Lapham S, McGue M, Mosing MA, Neiderhiser J, Nygaard M, Panizzon M, Prescott CA, Reynolds CA, Sachdev P, Whitfield KE (2019) IGEMS: The Consortium on interplay of genes and environment across multiple studies – an update. *Twin Research and Human Genetics, 22*, 493–499.
3. Pedersen DA, Larsen LA, Nygaard M, Mengel-From J, McGue M, Dalgård C, Hvidberg L, Hjelmborg J, Skytthe A, Holm NV, Kyvik KO, Christensen K (2019) The Danish Twin Registry: An updated overview. *Twin Research and Human Genetics, 22,* 499–507.
4. Christensen K, Holm NV, McGue M, Corder L, Vaupel JW (1999) A Danish population-based twin study on general health in the elderly. *Journal of Aging and Health, 11*, 49–64.
5. McGue M, Christensen K (2003) The heritability of depression symptoms in elderly Danish twins: Occasion-specific versus general effects. *Behavior Genetics, 33,* 83–93.
6. Lichtenstein P, Sullivan PF, Cnattingius S, Gatz M, Johansson S, Carlström E, Björk C, Svartengren M, Wolk A, Klareskog L, de Faire U, Schalling M, Palmgren J, Pedersen NL (2006) The Swedish Twin Registry in the third millennium: An update. *Twin Research and Human Genetics, 9,* 875–882.
7. Gatz M, Pedersen NL, Plomin R, Nesselroade JR, McClearn GE (1992) The importance of shared genes and shared environments for symptoms of depression in older adults. *Journal of Abnormal Psychology, 101,* 701–708.
8. McClearn GE, Johansson B, Berg S, Pedersen NL, Ahern F, Petrill SA, Plomin R (1997) Substantial genetic influence on cognitive abilities in twins 80 or more years old. *Science, 276*, 1560–1563.
9. Gold CH, Malmberg B, McClearn GE, Pedersen NL, Berg S (2002) Gender and health: A study of older unlike-sex twins*. Journal of Gerontology Series B, Psychological Sciences and Social Sciences 57*, S168–S176.
10. Neiderhiser JA, Reiss D, Lichtenstein P, Spotts EL, Ganiban J (2007) Father-adolescent relationships and the role of genotype-environment correlation. Journal of Family Psychology, 21, 560–571.
11. Sachdev PS, Lammel A, Trollor JN, Lee T, Wright MJ, Ames D, Wen W, Martin NG, Brodaty H, Schofield PR, OATS Research Team (2009) A comprehensive neuropsychiatric study of elderly twins: The Older Australian Twins Study. *Twin Research and Human Genetics, 12*, 573–582.
12. Sachdev PS, Lee T, Wen W, Ames D, Batouli AH, Bowden J, Brodaty H, Chong E, Crawford J, Kang K, Mather K, Lammel A, Slavin MJ, Thalamuthu A, Trollor J, Wright MJ, OATS Research Team (2013) The contribution of twins to the study of cognitive aging and dementia: The Older Australian Twins Study. *International Review of Psychiatry, 25*, 738–747.
13. Mosing MA, Zietsch BP, Shekar SN, Wright MJ, Martin NG (2009) Genetic and environmental influences on optimism and its relationship to mental and self-rated health: A study of aging twins. *Behavior Genetics, 39*, 597–604.
14. Finkel D, McGue M (1993) The origins of individual differences in memory among the elderly: A behavior genetic analysis. *Psychological Aging, 8*, 527–537.
15. Gatz M, Plassman BL, Tanner CM, Goldman SM, Swan GE, Chanti-Ketterl M, Walters EE, Butler DA (2019) The NAS-NRC Twin Registry and Duke Twins Study of Memory in Aging: An update. *Twin Research and Human Genetics, 22,* 757–760.
16. Kremen WS, Franz CE, Lyons MJ (2013) VETSA: The Vietnam Era Twin Study of Aging. *Twin Research and Human Genetics, 16*, 399–402.
17. Whitfield KE (2013) A registry of adult African American twins: The Carolina African American Twin Study of Aging. *Twin Research and Human Genetics, 16*, 476–480.
18. Whitfield KE, Brandon DT, Wiggins S, Vogler G, McClearn G (2003) Does intact pair status matter in the study of African American twins? The Carolina African American Twin Study of Aging. *Experimental Aging Research, 29*, 407–423.
19. Kendler KS, Thornton LM, Gilman SE, Kessler RC (2000) Sexual orientation in a US national sample of twin and nontwin sibling pairs. *American Journal of Psychiatry, 157*, 1843–1846.

Harmonized Depressive Symptoms Scale

Because different IGEMS studies administered different scales to assess depressive symptoms, it was necessary to establish a conversion table between different measures. Twins in the Older Australian Twins Study (OATS) and twins from the National Academy of Sciences/National Research Council (NAS/NRC) Twin Registry in the US were administered the 15-item Geriatric Depression Scale (GDS-15)[1].Twins in the Australian Over 50s study received the General Health Questionnaire (GHQ) [2]. Swedish studies and all other US studies were administered the 20-item Center for Epidemiologic Studies-Depression (CES-D)[3] scale, with a subset of Swedish twins and the Carolina African American Twins Study of Aging (CAATSA) receiving a shortened 11-item CES-D. Twins in the Danish studies were administered a modified version of the Cambridge Mental Disorders of the Elderly Examination (CAMDEX)[4]. We recruited a separate crosswalk sample who responded to all the different measures of depressive symptoms. Item response methods were used to co-calibrate the different measures, with the resulting harmonized depressive symptoms score expressed in CAMDEX units (range 16-46). This harmonized score permits pooling across IGEMS studies.[5]

References

1. Yesavage JA, Brink TL, Rose TL, Lum O, Huang V, Adey M, Leirer VO. (1982). Development and validation of a geriatric depression screening scale: a preliminary report. *Journal of Psychiatric Research*, *17*(1), 37–49.
2. Goldberg DP, Hillier VF. (1979). A scaled version of the General Health Questionnaire. *Psychological Medicine*, *9*(1), 139–145.
3. Radloff LS. (1977). The CES-D Scale: A self-report depression scale for research in the general population. *Applied Psychological Measurement, 1*(3), 385–401.
4. Roth M, Tym E, Mountjoy CQ, Huppert FA, Hendrie H, Verma S, Goddard R. (1986). CAMDEX. A standardised instrument for the diagnosis of mental disorder in the elderly with special reference to the early detection of dementia. *The British Journal of Psychiatry : the Journal of Mental Science*, *149*, 698–709
5. Gatz M, Reynolds CA, Finkel D, Hahn CJ, Zhou Y, Zavala C. (2015). Data harmonization in aging research: Not so fast. *Experimental Aging Research*, *41*(5), 475–495.

Fig. S1.

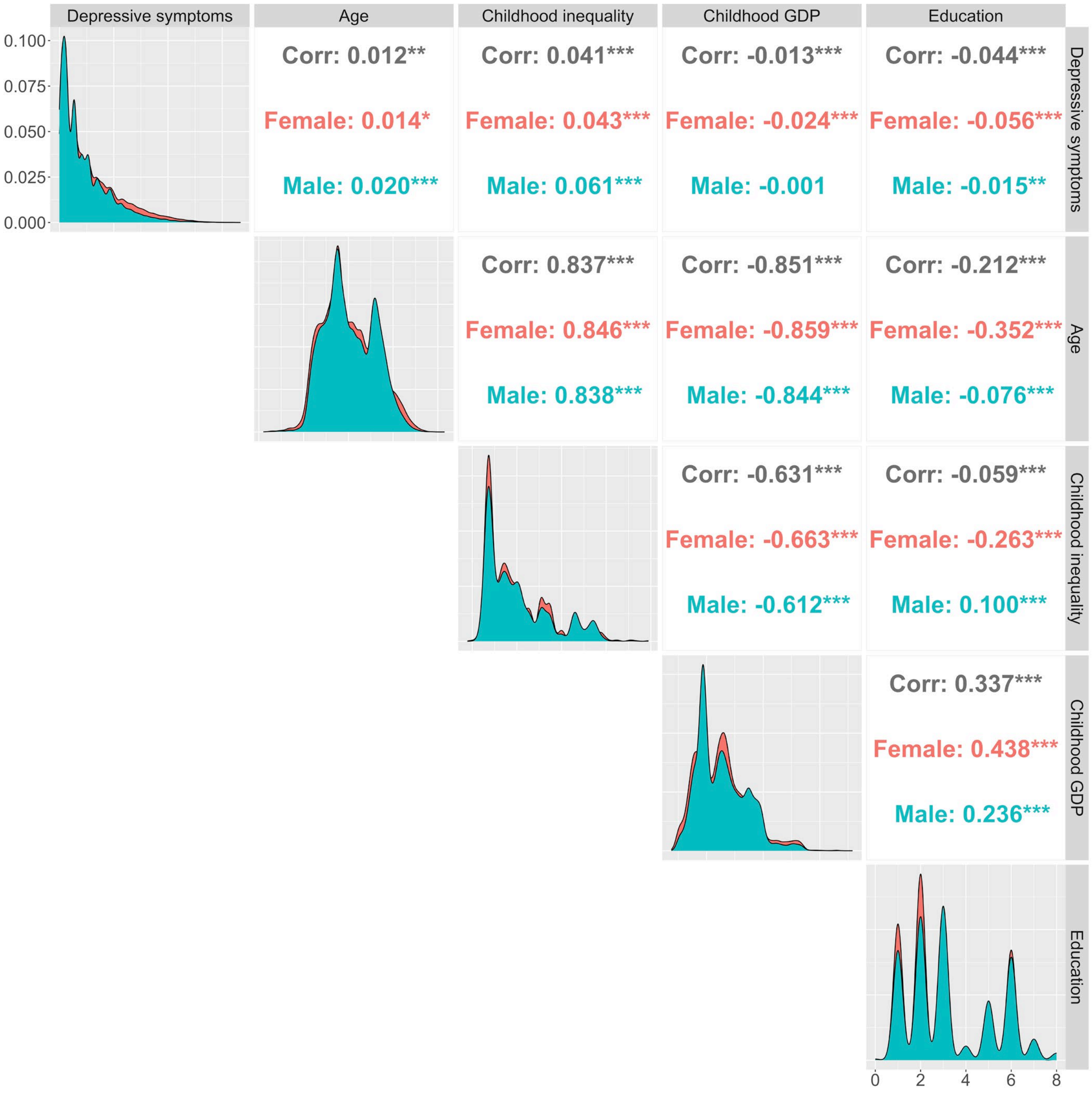

Supplemental Figure S1. Correlation matrix with distributions of the main variables of interest by sex.

Fig. S2.

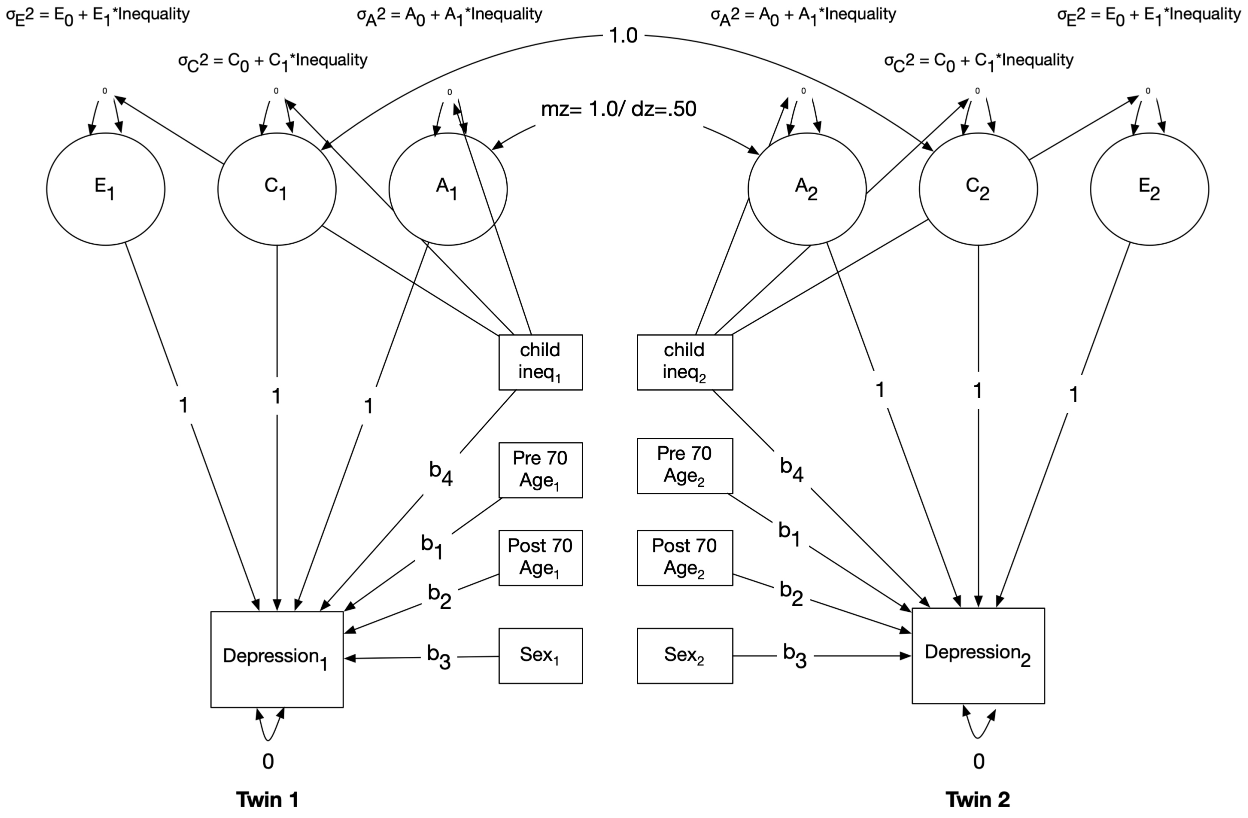

Supplemental Figure S2. The gene-by-environment model to examine whether childhood inequality moderates the variance additive genetic (A), common environmental (C), and nonshared environmental (E) contributions to depressive symptoms.

Fig. S3.

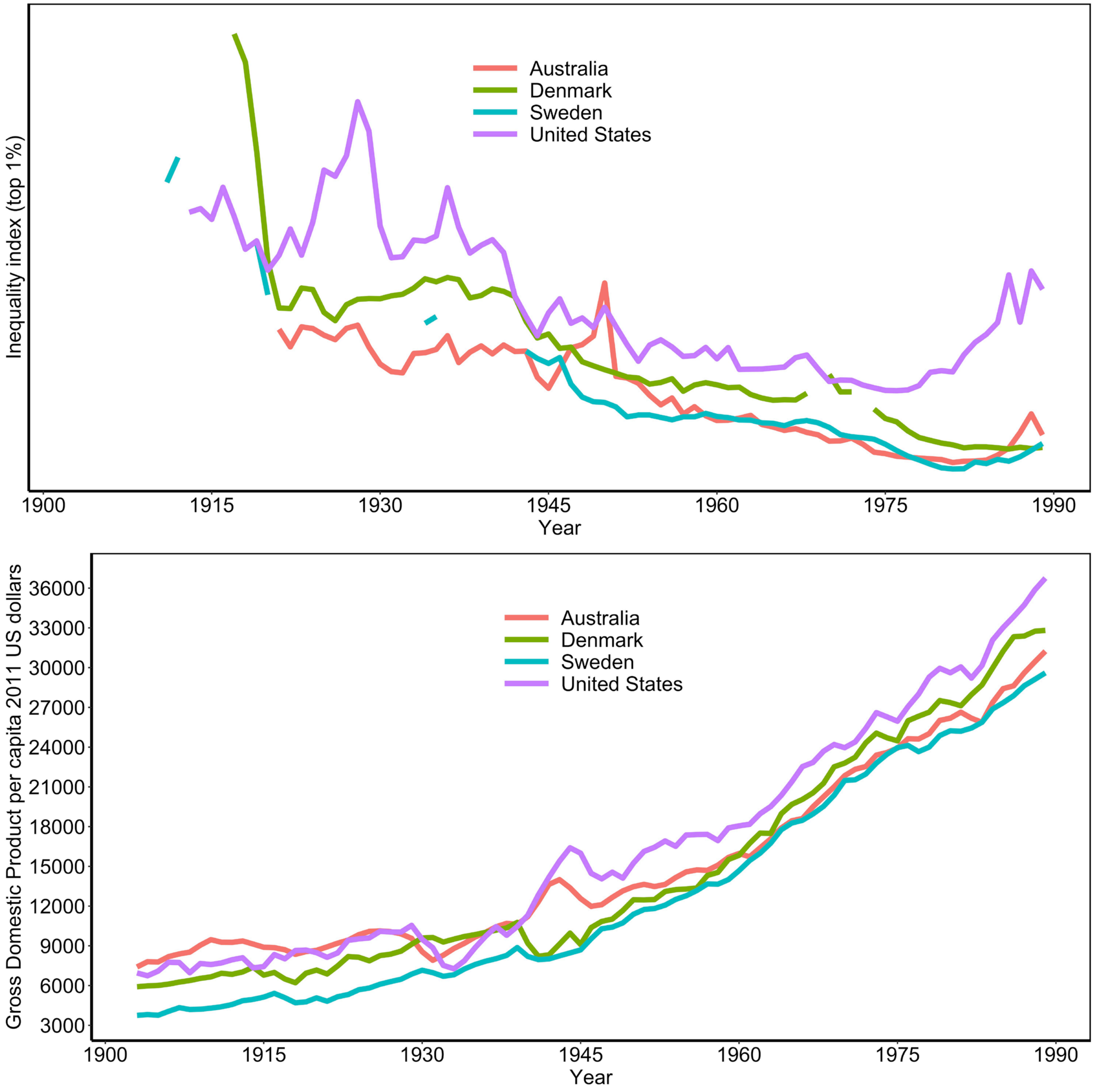

Supplemental Figure S3. Plot of income inequality (Panel A) and gross domestic product (GDP; Panel B) by year for the full range of childhood exposure to inequality.

Table S1. Sample descriptive statistics and comparisons between participants by polygenic index score availability (N=69,924)

Variable		Full Sample (N=69,924)<br>Mean (95% CI)	With genotyping (N=6,256)<br>Mean (95% CI)
Age at assessment		60.33 (60.24, 60.41)	63.62 (63.35, 63.88)
Zygosity %, (n)			
	DZ[a]	40% (40%, 41%)	37% (36%, 38%)
	MZ[b]	30% (30%, 30%)	45% (43%, 46%)
	OS[c]	30% (29%, 30%)	19% (18%, 20%)
Sex %, (n)			
	Female	51% (50%, 51%)	46% (45%, 47%)
	Male	49% (49%, 50%)	54% (53%, 55%)
Country of origin			
	Australia	4% (4%, 4%)	20% (19%, 21%)
	Denmark	23% (22%, 23%)	31% (30%, 33%)
	Sweden	63% (62%, 63%)	28% (27%, 29%)
	United States	10% (10%, 11%)	20% (19%, 21%)
Depressive symptoms[d]		21.13 (21.10, 21.16)	21.11 (21.00, 21.22)
Square root depressive symptoms		4.57 (4.57, 4.58)	4.57 (4.56, 4.58)
Calendar year at age 10		1949 (1949, 1949)	1945 (1945, 1945)
Calendar year at assessment		2000 (2000, 2000)	1999 (1998, 1999)
Top 1% index at age 10		9.75 (9.73, 9.78)	10.99 (10.94, 11.05)
GDP[e] at age 10		12.7 (12.6, 12.7)	12.1 (12.0, 12.2)
Educational attainment (ISCED[f])		3.18 (3.17, 3.20)	3.27 (3.22, 3.31)

[a] DZ = same sex dizygotic twin
[b] MZ = same sex monozygotic twin
[c] OS = opposite sex dizygotic twin
[d] Depressive symptoms = harmonized depressive symptoms score in Cambridge Mental Disorders of the Elderly Examination (CAMDEX) units
[e] GDP = Gross Domestic Product Per Capita (per thousands) in 2011 USA dollars
[f] ISCED = International Standard Classification of Education level

Table S2. Model fit statistics from the childhood income inequality, GDP, and PGI contributions to depressive symptoms.

Males (Parameters)	M1	CI	M2	CI	M3	CI	M4	CI	M5	CI	M6	CI
T1 [min(0,Age-70)]	-0.07	(-0.15 , 0.01)	0.16	(0.08 , 0.23)	0.04	(-0.05 , 0.12)	0.04	(-0.05 , 0.12)	0.04	(-0.04 , 0.12)	0.04	(-0.04 , 0.12)
T2 [max(0,Age-70)]	-0.06	(-0.22 , 0.10)	0.24	(0.10 , 0.38)	0.03	(-0.13 , 0.19)	0.03	(-0.13 , 0.20)	0.02	(-0.14 , 0.18)	0.03	(-0.14 , 0.19)
INEQ Ages 5 to 15	0.91	(0.55 , 1.28)	--	--	1.01	(0.64 , 1.39)	1.01	(0.64 , 1.38)	1.01	(0.63 , 1.39)	1.02	(0.66 , 1.38)
GDP Ages 5 to 15	--	--	0.31	(0.18 , 0.44)	0.34	(0.21 , 0.47)	0.34	(0.21 , 0.47)	0.34	(0.21 , 0.47)	0.34	(0.22 , 0.47)
MDD PGI	0.81	(0.37 , 1.25)	0.81	(0.36 , 1.25)	0.81	(0.37 , 1.25)	0.78	(0.29 , 1.28)	0.62	(0.17 , 1.08)	0.16	(-0.44 , 0.77)
INEQ x MDD PGI	--	--	--	--	--	--	0.03	(-0.26 , 0.32)	--	--	0.40	(0.05 , 0.75)
GDP x MDD PGI	--	--	--	--	--	--	--	--	0.10	(0.01 , 0.20)	0.19	(0.07 , 0.31)
Intercept	48.40	(47.45 , 49.35)	50.42	(49.62 , 51.21)	48.77	(47.81 , 49.73)	48.76	(47.81 , 49.72)	48.83	(47.87 , 49.79)	48.81	(47.87 , 49.74)
Residual Variance	95.55	(88.17 , 102.92)	95.46	(88.22 , 102.70)	94.41	(87.15 , 101.67)	94.41	(87.15 , 101.67)	94.21	(86.99 , 101.43)	94.03	(86.83 , 101.23)
Parameters	6		6		7		8		8		9	
Log-Likelihood	-12461.0		-12459.5		-12440.9		-12440.9		-12437.3		-12434.1	
AIC	24934.1		24930.9		24895.8		24897.7		24890.6		24886.2	
BIC	24970.8		24967.6		24938.6		24946.7		24939.6		24941.3	
MLR Scaling Correction Factor	1.51		1.53		1.52		1.51		1.52		1.47	
Comparison Model	M3		M3		--		M6		M6		M3	
∆-2LL Scaling Correction	1.56		1.47		--		1.14		1.11		1.30	
∆−2LL Adjusted Chisq	25.79		25.24		--		11.93		5.87		10.48	
∆ parameters	1		1		--		1		1		2	
p	3.80E-07		5.07E-07		--		6.00E-04		1.54E-02		5.30E-03	

Females (Parameters)	M1	CI	M2	CI	M3	CI	M4	CI	M5	CI	M6	CI
T1 [min(0,Age-70)]	0.06	(0.00 , 0.13)	-0.11	(-0.18 , -0.04)	--	--	0.06	(0.00 , 0.13)	-0.11	(-0.18 , -0.04)	--	--
T2 [max(0,Age-70)]	-0.17	(-0.30 , -0.03)	-0.15	(-0.26 , -0.04)	--	--	-0.17	(-0.30 , -0.03)	-0.15	(-0.26 , -0.04)	--	--
INEQ Ages 5 to 15	0.91	(0.62 , 1.19)	--	--	--	--	0.91	(0.62 , 1.19)	--	--	--	--
GDP Ages 5 to 15	--	--	-1.27	(-1.47 , -1.07)	--	--	--	--	-1.27	(-1.47 , -1.07)	--	--
MDD PGI	0.70	(0.20 , 1.20)	0.60	(0.12 , 1.08)	--	--	0.67	(0.14 , 1.21)	0.55	(0.03 , 1.06)	--	--
INEQ x MDD PGI	--	--	--	--	--	--	0.02	(-0.17 , 0.21)	--	--	--	--
GDP x MDD PGI	--	--	--	--	--	--	--	--	-0.02	(-0.18 , 0.13)	--	--
Intercept	50.08	(49.32 , 50.85)	48.11	(47.30 , 48.92)	--	--	50.08	(49.32 , 50.85)	48.11	(47.30 , 48.92)	--	--
Residual Variance	97.77	(89.72 , 105.83)	93.40	(85.50 , 101.31)	--	--	97.77	(89.71 , 105.83)	93.40	(85.49 , 101.31)	--	--
Parameters	6	--	6	--	--	--	7	--	7	--	--	--
Log-Likelihood	-10711.51	--	-10645.52	--	--	--	-10711.48	--	-10645.46	--	--	--

AIC	21435.03	--	21303.04	--	--	--	21436.97	--	21304.92	--	--	--
BIC	21470.83	--	21338.84	--	--	--	21478.74	--	21346.7	--	--	--
MLR Scaling Correction Factor	1.48	--	1.44	--	--	--	1.45	--	1.4	--	--	--
Comparison Model	--	--	--	--	--	--	M1	--	M2	--	--	--
Δ-2LL Scaling Correction	--	--	--	--	--	--	1.29	--	1.22	--	--	--
Δ–2LL Adjusted Chisq	--	--	--	--	--	--	0.04	--	0.09	--	--	--
Δ parameters	--	--	--	--	--	--	1	--	1.0	--	--	--
p	--	--	--	--	--	--	8.32E-01	--	7.60E-01	--	--	--

MDD PGI = polygenic index for major depressive disorder, INEQ = income inequality
Parameters = number of freely estimated parameters from the model
MLR scaling correction factor = MPLUS correction factor for the MLR estimator. The correction factor is needed to conduct model comparison of nested models when using the MLR estimator.
Δ-2LL = the difference in 2 times the negative log-likelihood in the two nested models that were compared.
Δ parameters = the difference in parameters from the two nested models that were compared.
p = the probability value from the Δ-2LL by Δpara test. A p value of less than .05 is suggestive of a significant difference in model fit between the two models that were compared.
AIC = Akaike Information Criterion
BIC = Bayesian Information Criterion

Table S3. Twin correlations by MZ (8,192 complete pairs), DZ (9,686 complete pairs), and OS (7,342 complete pairs).

Variable		r	95% Confidence Interval
Zygosity			
	MZ[a]	0.345	[0.326, 0.364]
	DZ[b]	0.187	[0.168, 0.206]
	OS[c]	0.136	[0.113, 0.158]

[a] MZ = same sex monozygotic twin
[b] DZ = same sex dizygotic twin
[c] OS = opposite sex dizygotic twin

Table S4. Model fit statistics from the childhood income inequality moderation of the genetic and environmental contributions to depressive symptoms.

Model	Description	Log-Likelihood	Parameters	MLR Correction Factor	Comparison model	Δ-2LL	Δ para	p	AIC	BIC
1	ACE inequality moderation	-243597.24	11	.82	na	na	na	na	487216	487312
2	AE Inequality moderation	-243597.23	9	.95	1	0.01	2	0.999	487212	487290
3	AE drop A1moderation parameter	-243601.40	8	.95	2	8.78	1	0.003	487219	487288
4	AE drop E1 moderation parameter	-243597.64	8	.94	2	0.80	1	0.645	487211	487281

Parameters = number of freely estimated parameters from the model

MLR correction factor = MPLUS correction factor for the MLR estimator. The correction factor is needed to conduct model comparison of nested models when using the MLR estimator.

Δ-2LL = the difference in 2 times the negative log-likelihood in the two nested models that were compared.

Δ para = the difference in parameters from the two nested models that were compared.

p = the probability value from the Δ-2LL by Δpara test. A p value of less than .05 is suggestive of a significant difference in model fit between the two models that were compared.

AIC = Akaike Information Criterion

BIC = Bayesian Information Criterion
